# Body-size structure of Central Iberian mammal fauna reveals semidesertic conditions during the middle Miocene Global Cooling Event

**DOI:** 10.1371/journal.pone.0186762

**Published:** 2017-10-26

**Authors:** Iris Menéndez, Ana R. Gómez Cano, Blanca A. García Yelo, Laura Domingo, M. Soledad Domingo, Juan L. Cantalapiedra, Fernando Blanco, Manuel Hernández Fernández

**Affiliations:** 1 Departamento de Paleontología, Facultad de Ciencias Geológicas, Universidad Complutense de Madrid, Madrid, Spain; 2 Departamento de Geología Sedimentaria y Cambio Medioambiental, Instituto de Geociencias (UCM, CSIC), Madrid, Spain; 3 Transmitting Science, Barcelona, Spain; 4 Institut Català de Paleontologia Miquel Crusafont, Universitat Autónoma de Barcelona, Cerdanyola del Vallès, Spain; 5 Departamento de Paleobiología, Museo Nacional de Ciencias Naturales (CSIC), Consejo Superior de Investigaciones Científicas, Madrid, Spain; 6 Earth and Planetary Sciences Department, University of California Santa Cruz, Santa Cruz, United States of America; 7 Departamento de Ecología Evolutiva, Estación Biológica de Doñana (CSIC), Sevilla, Spain; 8 Museum für Naturkunde, Leibniz-Institut für Evolutions und Biodiversitätsforschung, Berlin, Germany; Royal Belgian Institute of Natural Sciences, BELGIUM

## Abstract

We developed new quantitative palaeoclimatic inference models based on the body-size structure of mammal faunas from the Old World tropics and applied them to the Somosaguas fossil site (middle Miocene, central Iberian Peninsula). Twenty-six mammal species have been described at this site, including proboscideans, ungulates, carnivores, insectivores, lagomorphs and rodents. Our analyses were based on multivariate and bivariate regression models correlating climatic data and body-size structure of 63 modern mammal assemblages from Sub-Saharan Africa and the Indian subcontinent. The results showed an average temperature of the coldest month higher than 26°C for the Somosaguas fossil site, a mean annual thermal amplitude around 10°C, a drought length of 10 months, and an annual total precipitation greater than 200 mm per year, which are climate conditions typical of an ecotonal zone between the savanna and desert biomes. These results are congruent with the aridity peaks described over the middle Aragonian of Spain and particularly in the local biozone E, which includes Somosaguas. The aridity increase detected in this biozone is associated with the Middle Miocene Global Cooling Event. The environment of Somosaguas around 14 Ma was similar to the current environment in the Sahel region of North Africa, the Horn of Africa, the boundary area between the Kalahari and the Namib in Southern Africa, south-central Arabia, or eastern Pakistan and northwestern India. The distribution of modern vegetation in these regions follows a complex mosaic of plant communities, dominated by scattered xerophilous shrublands, semidesert grasslands, and vegetation linked to seasonal watercourses and ponds.

## Introduction

Globally, the middle Miocene marked the transition from the globally warmer and more humid conditions that characterized the early Miocene and great part of the Palaeogene towards a more arid, seasonal and heterogeneous world. The so called “Middle Miocene Global Cooling Event” (MMGC) took place around 14 Ma, and is linked to the reestablishment of the Eastern Antarctic ice sheet [1–3]. The MMGC caused a profound shift in terrestrial ecosystems [4]. In particular, it promoted the development of semi-arid landscapes—broadly classified as savannas—and the appearance of a new set of mammalian communities adapted to the newly created environments.

Mammals are very sensitive to climatic perturbations [5, 6–8] which, in conjunction with a fairly continuous and well studied fossil record, makes them one of the best proxies to detect climate change in continental environments [9, 10, 11]. In the last decades, numerous palaeoclimatic and palaeoenvironmental inference methods have been proposed for mammalian fossil sites based on shifts in feeding and drinking habits of herbivorous mammals [12–15], locomotor adaptations [16–18], and different aspects of the community structure [9, 19–26]. However, few of these studies develop quantitative approaches to infer palaeoclimatic conditions [27–34], as a result of the complexity of calculating absolute values of past climatic variables due to the interaction of multiple changing sets of environmental conditions in space and time. For example, to set a limit between arid and humid conditions has proved to be particularly difficult in palaeoclimatic studies [35] because there is a continuous gradient in aridity across several biomes, which is far from linear and affects plant and animal species in transition areas in different ways. Besides, the relationship between seasonality of moisture and thermal seasonality determines the intensity of aridity, which is also conditioned by rainfall timing [36]. Recurring winds and mountain ranges also disrupt the trajectory of cyclones, creating “rain shadow” effects [37]. Finally, soil characteristics may contribute to increasing or decreasing the effects of aridity by means of the variation in their water-holding capacity [38].

One of such complex transitions is the shift from tropical forests and woodlands to savannas and deserts [11, 35, 39]. The transition from tropical forest to savanna occurs where the availability of moisture and nutrients throughout the year is inadequate to sustain closed forests with well developed shrub layers, while semideserts and deserts appear when moisture is not able to maintain a continuous vegetation cover. Nevertheless, the distribution of vegetation in tropical areas, while closely related to current climate, is also influenced by soil conditions, by the short-term effects of natural disturbances, and by the long-term geomorphological changes in the landscape as well as by climatic changes over geological time [40, 41]. The term “savanna” has been usually used in a very broad sense to embrace all wooded grasslands between the tropical rainforests and the deserts, including a continuum of structural forms ranging from closed-canopy stratified woodland to open treeless grasslands. These have been variously termed woodland, parkland, savanna woodland, dry forest, moist or dry savanna, low tree and shrub community, thicket, bushveld, thorn savanna, tree savanna, tropical steppe, etc. All these categories of savanna are determined by the height and spacing of trees, shrubs, and grasses, which is influenced mainly by soil moisture conditions. Within given climatic limits water availability depends on the bedrock geology or on superficial deposits and, consequently, various categories of savanna vegetation occur in juxtaposition and exhibit complex distributions related to soil conditions [42–44].

In this context, the Somosaguas vertebrate fossil site, located at the Madrid Basin (Spain), has provided unprecedented evidence of the influence of MMGC and the development of open landscapes at the community scale, by means of faunal [24, 39, 45–47], mineralogical [48, 49] and isotopic analyses [12, 13].

The rationale of this study consists on extending our proxy-based palaeoclimatic inferences in this fossil site to additional quantitative analyses, since results can be contrasted with those previous studies. The main aim of this work was to parameterize climatic variables for the Somosaguas fossil site based on the body-size structure of mammalian faunas, which is intimately related to climate and vegetation [10, 19, 20, 50–53]. First, we used the variation of the body-size community structure of different modern mammalian associations from Africa and Asia in relation to climatic variables (mean annual temperature, mean temperature of the coldest and warmest months, mean annual thermal amplitude, annual total precipitation, and drought length) for the development of multi- and bivariate statistical models in order to quantify these climatic variables. Then we applied the inference models to the Somosaguas fossil site body-size structure and obtained the climatic variables inferred for the middle Miocene of Somosaguas. The accuracy and congruence of these results were assessed by means of comparison with previous quantitative and qualitative inferences derived from the isotopic, mineralogical and faunal record of the Somosaguas fossil site. We conclude by describing how the climate and vegetation of the middle Miocene of Somosaguas would have been.

## Material and methods

### The Somosaguas fossil site

**Fig 1:**
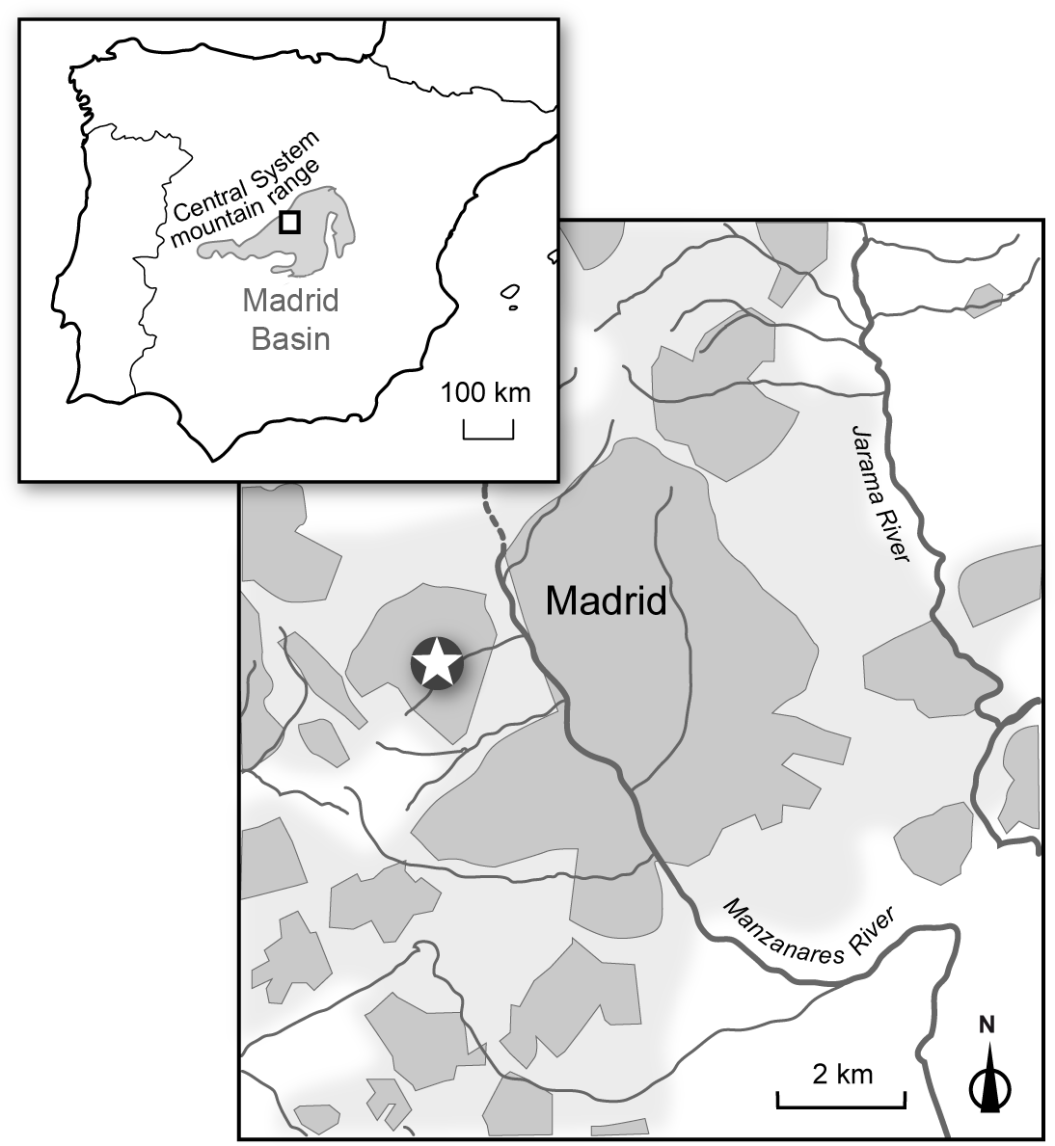
General situation of the Madrid Basin within the Iberian Peninsula (upper left inset) and detailed geographical situation of the Somosaguas fossil site (marked with a star) to the west of the city of Madrid. Modified from García Yelo et al. [39].

The Somosaguas fossil site is located in central Spain, in the deposits belonging to the Madrid Basin (Fig 1), which is filled by Cenozoic continental deposits originated in the mountain ranges surrounding the basin [54]. The mineralogy of the Madrid Basin deposits reflects the composition of these mountain ranges, which are mainly composed of granitic and metamorphic rocks (Central System mountain range and the Toledo Mountains) and sedimentary rocks (Iberian and Altomira Ranges). The Madrid Basin was an endorheic sedimentary basin during the early and middle Miocene, occupied by central lacustrine and palustrine systems fringed by alluvial fans and fluvial distributary facies [54]. Lacustrine environments such as the ones registered in the Intermediate Unit of the central Madrid Basin are relatively common in modern arid and semiarid environments. Typical examples can be found, for instance, in Etosha (Namibia) or the Magadi-Natron Basin (Kenya, Tanzania). During the late Miocene to the early Pliocene, the Madrid Basin became an exorheic basin. Therefore, the deposits are divided in three units. The Lower Unit (Ramblian to Lower Aragonian, ~25–19 Ma) is composed of lacustrine deposits in the central areas and detritic materials towards the borders of the basin (fluvial systems and alluvial fans). The Intermediate Unit (Middle Aragonian to Lower Vallesian, ~19–10 Ma) has the same concentric model as the Lower Unit, but the detritic deposits are more extended as a consequence of the reactivation of the Central System uplift. Finally, the Upper Unit (Upper Vallesian to Turolian, ~ 10–5 Ma) corresponds to the change from endorheic to exorheic basin, and is composed of terrigenous deposits accumulated by fluvial systems and carbonate sediments that accumulated in a fresh-water fluviolacustrine system [54].

The Somosaguas deposits belong to the Intermediate Unit of the Madrid Basin [55], which records a change from sulfate to carbonate sedimentation, followed by significant progradation of detrital materials from the northern and western borders toward the centre of the basin [49, 54]. Detrital materials were deposited by alluvial fan systems and consist of conglomerates and coarse-grained arkosic sandstones in their proximal zones, while in middle zones there are finer-grained arkosic sandstones intercalated with lutites; in distal zones the clayey levels dominate, with intercalations of micaceous sands, gypsum and carbonates [56]. Micaceous sands have been related to transitional facies between the proximal and central areas of the basin, where levels of carbonates were deposited in marsh or shallow lake environments [57].

Three stratigraphic levels (T1, T2 and T3) have been recognized in the Somosaguas area, corresponding to terrigenous materials derived mainly from the Central System, and representing different sedimentary environments within alluvial fans [49, 58, 59]. Vertebrate fossils have only been found in the lower and upper intervals (T1 and T3), while no such fossils have been discovered in the intermediate interval (T2). On account of the composition of the fossiliferous assemblage, we distinguish between two main sites, South and North Somosaguas. South Somosaguas corresponds to the lower interval (T1), formed by clayey arkose sediments that were extracted and processed in order to obtain small-mammal remains, and has been interpreted as the result of mud-flow deposits [58, 60]. Due to the low energy associated with these mud-flow deposits very few large-mammal remains have been found. Large remains are usually associated with the coarser grain deposits in the area [45], such as the ones in North Somosaguas, where we found a silty-clay matrix and poorly sorted coarse arkosic sandstones and fine conglomerates within, which difficult the extraction of small-mammal remains. This site corresponds to the upper interval (T3) and has been interpreted as the result of successive debris-flow deposits.

The Somosaguas locality stands out as one of the most studied middle Miocene fossil vertebrate assemblages of the Madrid Basin, gathering between the two sites (North and South Somosaguas) a total of 32 species of vertebrates, 26 of which are mammals [46, 47, 61–64]. The faunal data of the middle Miocene Somosaguas vertebrate association can provide valuable information about the palaeoecology of the recovered taxa, which in turn can be used as a proxy to unveil palaeoclimatic and palaeoenvironmental conditions existing in the terrestrial record during an episode of profound global change as the Middle Miocene Cooling Event.

Luis & Hernando [60] included the site in Biozone E of the middle Aragonian (middle Miocene), which has been dated around 14 Ma by means of magnetostratigraphy and biostratigraphy covering a total time span of ca. 250,000 years [64, 65].

Therefore, faunal, isotopic and sedimentary analyses of the Somosaguas record suggest that, during the Miocene, a tropical or subtropical savanna stretched around a seasonal lake, in an increasingly cold and arid palaeoclimatic environment [12, 24, 39, 45–48].

### Body-size structure

The body-size structure of mammalian communities has been proposed as an adequate parameter of community description [50–52, 66–68], which is associated with the concept of community convergence [69]. Convergence as a phenomenon is well known. Since animal and plant traits are shaped by natural selection, analogous morphological and functional adaptations to similar environmental challenges have often arisen independently in unrelated lineages [70], giving rise to species that are ecologically equivalent. In this sense, the term convergent evolution is a concept in evolutionary biology applied at the species level with examples ranging over a wide spectrum of taxa and habitats. Because environments select organisms on the basis of their adaptations, and these are expressed simultaneously in their morphology, physiology and ecology, then different assemblages of species inhabiting similar environments should also have predictable properties. Thus, convergent evolution may also extend to patterns of organization and structure at the community level [71, 72], and may produce similarities in resource utilization and diversity in geographically distinct communities dependent on similar environments [73–76]. Therefore, since communities developing under similar environmental conditions have similar community structures, it makes it possible to implement palaeoclimatic studies at the community scale [19, 20, 23, 35, 39, 53, 77, 78].

Due to the rarity of species of the orders Carnivora and Chiroptera in fossil sites of fluvial, alluvial or lacustrine origin, we excluded bats and carnivorans from the dataset for the study of the body-size structure of mammalian communities [10, 20, 39]. Each species was assigned to one body size category in order to better represent the body-size structure of each community (Table 1). Andrews et al. [19] defined eight weight subdivisions of living mammals, which have been subsequently reviewed and modified on several occasions [22, 23, 27, 35, 39, 79–84]. Moreover, to avoid the influence of species richness in our comparison among modern communities of different biomes as well as with the Somosaguas Miocene community, the number of species in each category was transformed into percentage of species.

**Table 1:** Body size categories defined to determine the body-size structure of the mammalian communities.

Category ^a^	Weight range (g)
A	0–100
B	100.1–1000
C	1000.1–10000
D	10000.1–45000
E	45000.1–90000
F	90000.1–180000
G	180000.1–360000
H	> 360000

^a^ After Hernández Fernández et al. [35], García Yelo et al. [39], and Fernández-Jalvo et al. [82]

We used the allometric equations developed by Creighton [85] and Legendre [10], which relate body mass and the surface area of the first lower molar, in order to categorize Somosaguas mammal body sizes (Table 2). Since direct measurement of the surface of the first lower molar was not always possible due to preservation/taphonomic issues, body masses of some species were taken from the literature [22, 47, 86]. In the case of the equid *Anchitherium*, body mass was estimated from the equations developed by Alberdi et al. [87].

**Table 2:** Faunal list of the terrestrial non-carnivorans recorded in the Somosaguas fossil site and the body size category assigned to them.

Order	Family	Species	Body size category ^a^
Artiodactyla	Bovidae	*Tethytragus* sp.	E
	Cervidae	*Heteroprox* sp.	D
	Moschidae	*Micromeryx* nov. sp. (According to Sánchez et al. [88])	C
	Tragulidae	*Dorcatherium* sp. cf. *D*. *crassum*	D
	Suidae	*Retroporcus complutensis*	F
Eulipotyphla	Erinaceidae	*Galerix exilis*	A
	Erinaceidae	*Amphaechinus* sp.	C
	Soricidae	*Miosorex* sp. cf. *M*. *grivensis*	A
Lagomorpha	Ochotonidae	*Lagopsis penai*	B
	Ochotonidae	*Prolagus* sp. cf. *P*. *oeningensis*	A
Perissodactyla	Equidae	*Anchitherium* sp. cf. *A*. *cursor*	E
	Rhinocerotidae	*Prosantorhinus douvillei*	H
Proboscidea	Gomphoteriidae	*Gomphotherium angustidens*	H
Rodentia	Cricetidae	*Cricetodon soriae*	B
	Cricetidae	*Democricetodon lacombai*	A
	Cricetidae	*Democricetodon larteti*	A
	Cricetidae	*Megacricetodon collongensis*	A
	Gliridae	*Armantomys tricristatus*	B
	Gliridae	*Microdyromys aff*. *M*. *monspeliensis*	A
	Gliridae	*Microdyromys nov*. *sp*. (According to Hernández-Ballarín & Peláez-Campomanes [64])	A
	Sciuridae	*Heteroxerus rubricati*	A
	Sciuridae	*Heteroxerus grivensis*	B
			

^a^ Categories A–H as in Table 1

### Extant faunas

**Fig 2:**
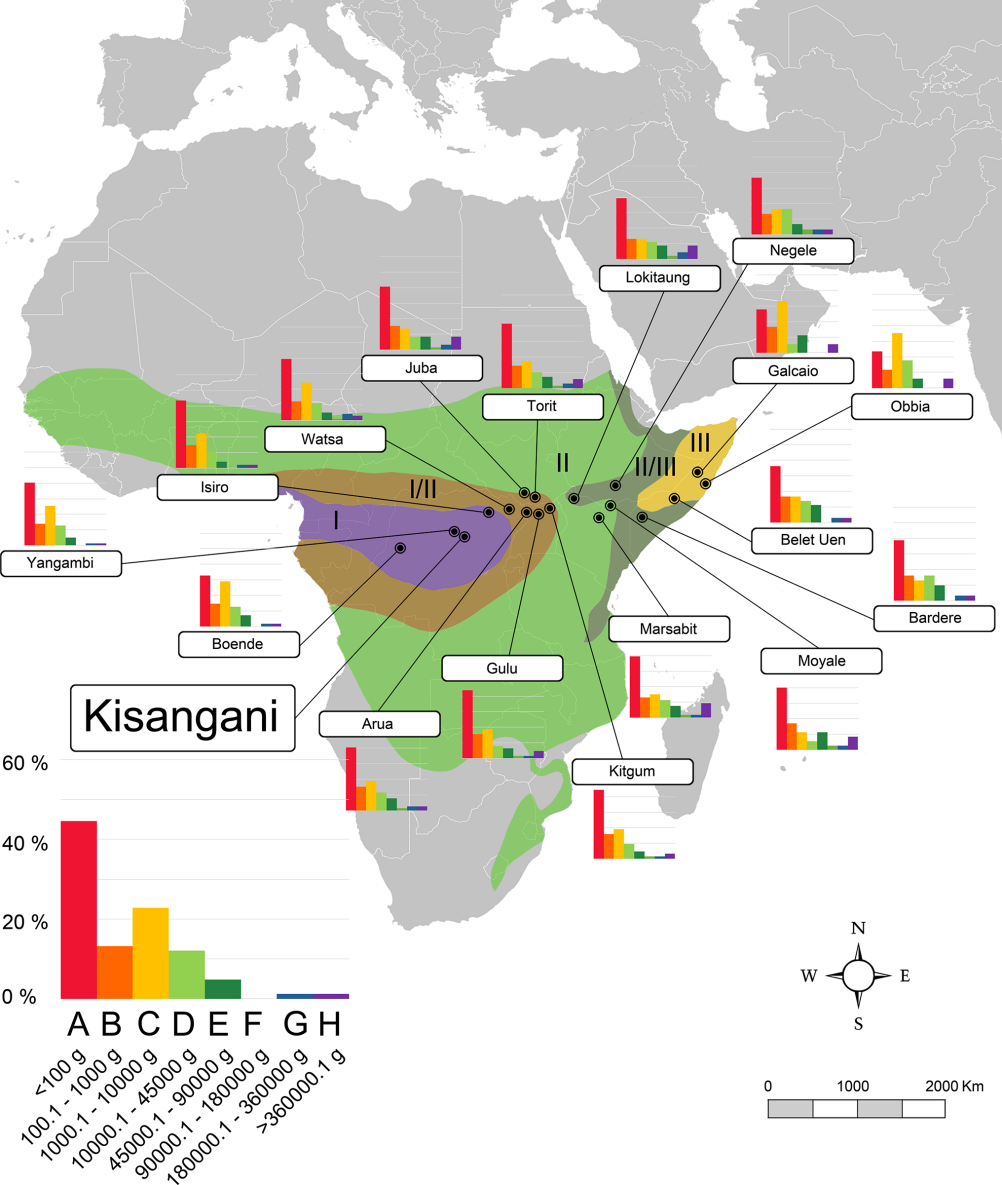
Map of Africa showing the localities of the Equatorial Africa transect (transect 1) included in this work and the body-size structure of each one showed as histograms (species percentages vs. body size categories). Biomes described by Walter (1985) are also shown: I, evergreen tropical rain forest (purple); I/II, semi-evergreen tropical forest (brown); II, tropical deciduous woodland (light green); II/III, savanna (dark green); III, subtropical desert (yellow). A larger graph for one locality is shown for information on the axes values. A–H, body size categories as in Table 1. Data for each locality are shown in S1 Table.

**Fig 3:**
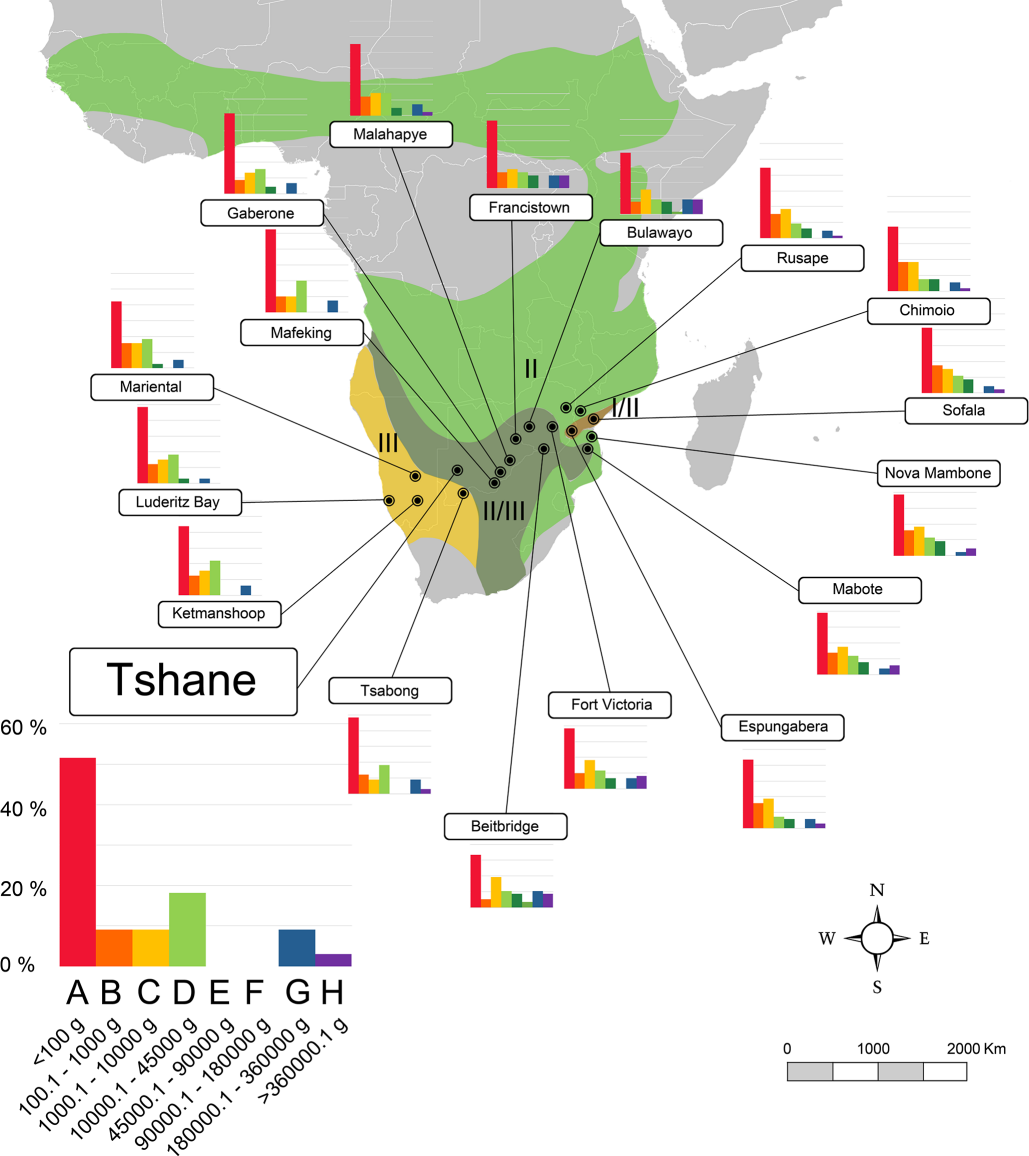
Map of Africa showing localities of the Southern Africa transect (transect 2) used in this work and the body-size structure of each one (species percentages vs. body size categories). Biomes as in Fig 2. A larger graph for one locality is shown for information on the axes values. A–H, body size categories as in Table 1. Data for each locality are shown in S1 Table.

**Fig 4:**
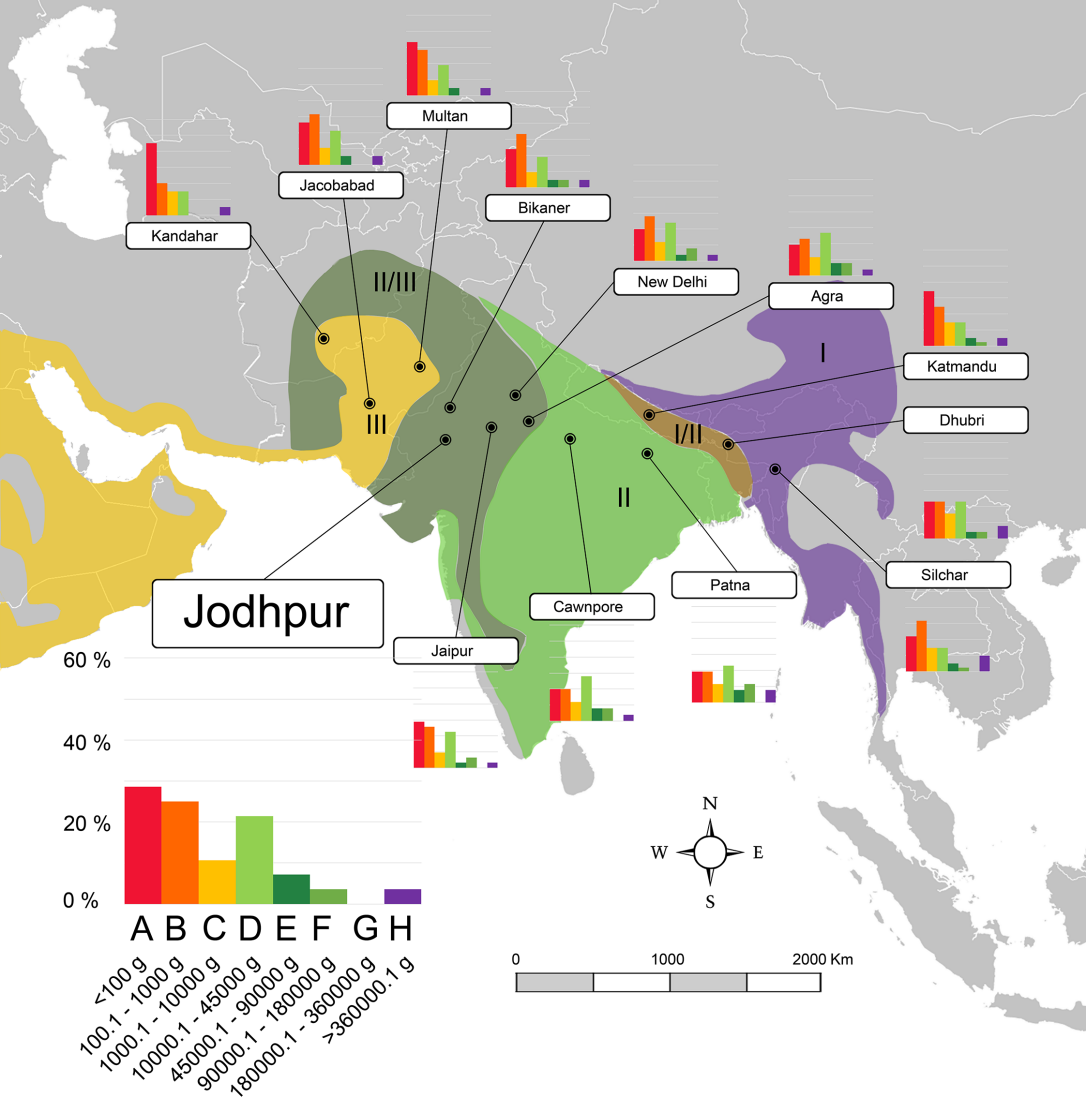
Map of South Asia showing the localities of the northern Indian subcontinent transect (transect 3) used in this work and the body-size structure of each one (species percentages vs. body size categories). Biomes as in Fig 2. A larger graph for one locality is shown for information on the axes values. A–H, body size categories as in Table 1. Data for each locality are shown in S2 Table.

**Fig 5:**
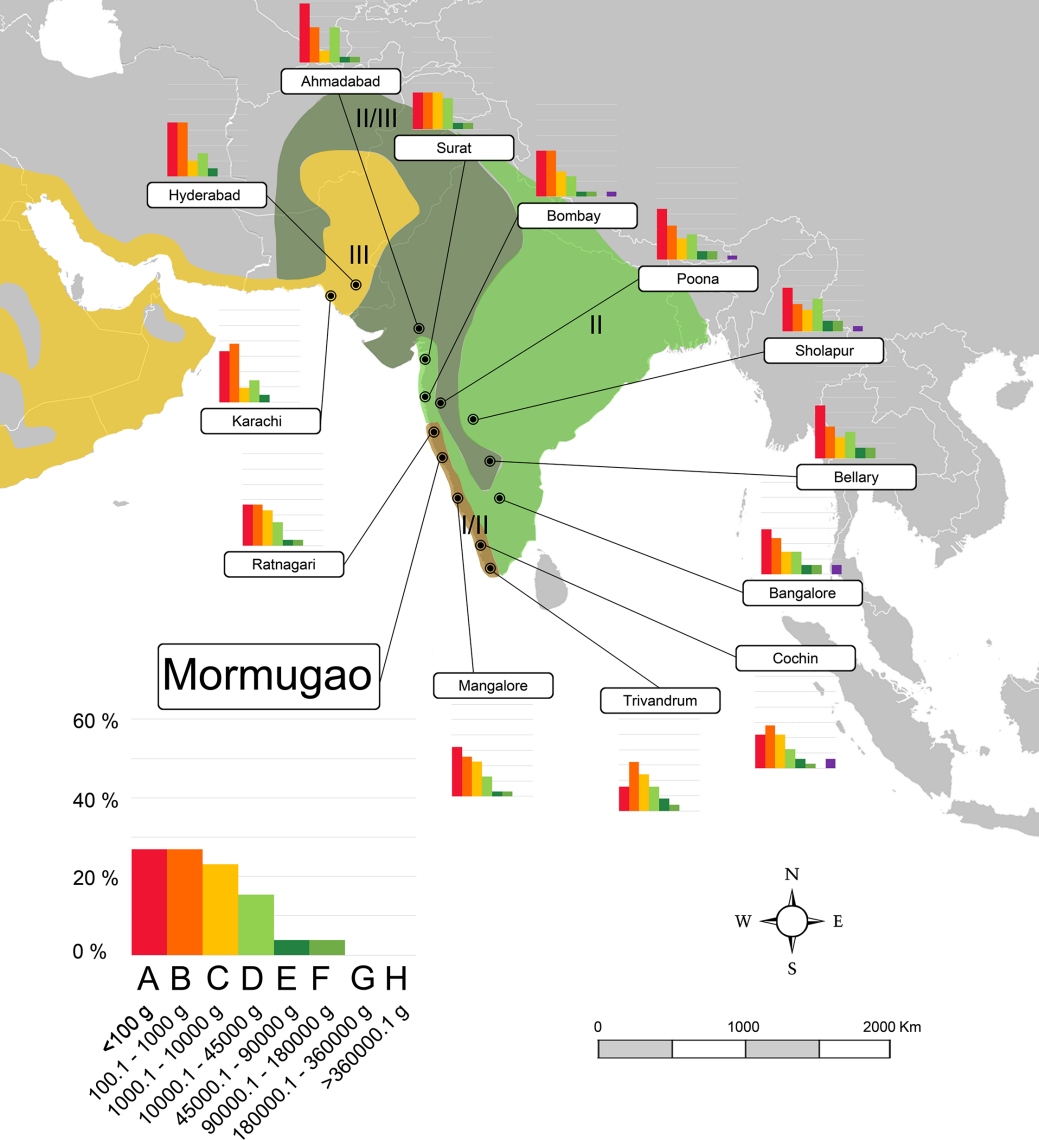
Map of South Asia showing the localities of the western India and southern Pakistan transect (transect 4) used in this work and the body-size structure of each one (species percentages vs. body size categories). Biomes as in Fig 2. A larger graph for one locality is shown for information on the axes values. A–H, body size categories as in Table 1. Data for each locality are shown in S2 Table.

In order to obtain quantitative palaeoenvironmental variables, the body-size structure of Somosaguas was compared with data compiled from 63 extant mammalian communities from the Old World tropics, containing both faunal and climatic information collected from the literature (S1 and S2 Tables). We selected localities from this area because they have larger historical and evolutionary relationships with the Miocene faunas from Spain than those from other continents [89]. Since previous works inferred a tropical or sub-tropical environment for Somosaguas [12, 24, 39, 45–48], the localities were selected in order to represent all the possible biomes and ecotones following a climatic gradient within tropical lowlands as defined by Walter [36], from the evergreen equatorial forest (I in Figs 2–5), through semi-evergreen tropical forest (I/II), tropical deciduous woodland (II), and savanna (II/III) to the tropical desert (III).

In order to include as much climatic and faunal variability as possible, the localities were distributed in four different transects (Figs 2–5) including all those biomes, across Equatorial Africa (transect 1, 18 localities), Southern Africa (transect 2, 18 localities), the northern area of the Indian subcontinent (transect 3, 13 localities), and western India and southern Pakistan (transect 4, 14 localities). Data of the climatic variables (summarized in S3 Table) of every locality were collected from Meteorological Office [90, 91] and Rivas-Martínez et al. [92]. The faunal list of each locality was compiled from Corbet et al. [93], Kingdon [94] and IUCN [95] following taxonomic allocations from Wilson et al. [96]. Introduced mammals were not included in the database. The information of body masses for living taxa were compiled from the PanTHERIA database [97]. For those few extant taxa for which no mass data were found, masses were estimated based on the average mass of other members of the same genus [35, 53, 77]. Finally, the body-size structure of each modern community was calculated using the methodology previously commented.

### Quantitative inference of climatic variables

**Fig 6:**
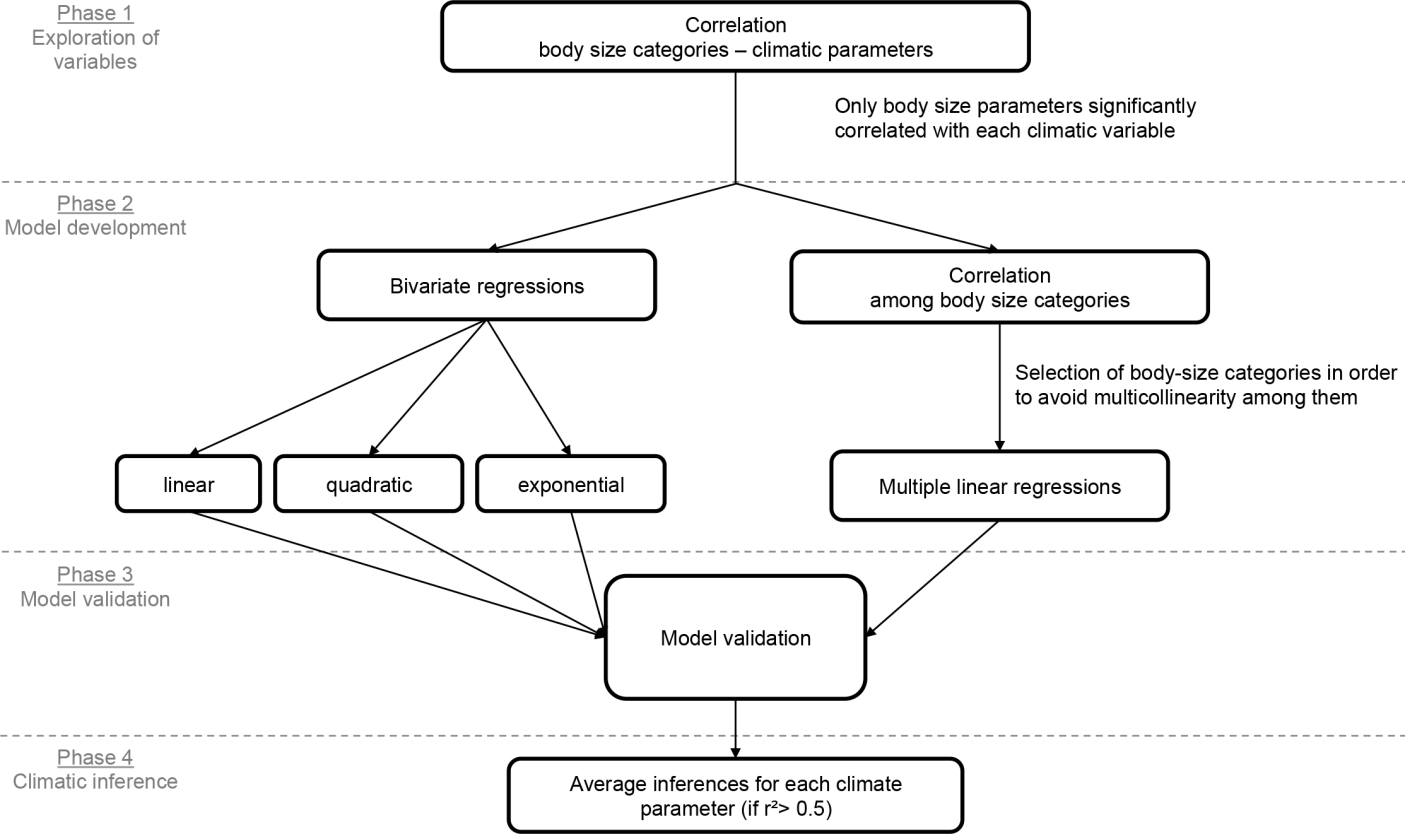
Flow chart showing the analytical processes used in order to obtain quantitative inferences for each climatic parameter.

In order to infer climatic values for the Miocene assemblage of Somosaguas, we computed ordinary least squares (OLS) regressions using the categories of the body-size structure as independent variables, and each of the climatic variables as dependent variables. Our approach included several successive phases in order to develop independent models for each climatic variable in both multivariate and bivariate frameworks as well as, finally, a climatic inference for Somosaguas (Fig 6).

The purpose of multiple regression analysis is to infer a single variable from a set of independent variables, providing a powerful method to analyze multivariate data. Nevertheless, many problems tend to arise when there are many independent variables in a multiple regression equation [98, 99]. Thus, the number of independent variables in the equation should be limited, and variables that do not contribute very much to explain the variance in the dependent variable (i.e., to the total r^2^) must be eliminated.

Since this work focuses on building models to infer values of different climatic variables, we selected a subset of independent variables (body size categories) that explain variation in the response of the dependent variables (each climatic parameter). Therefore, only body size categories that showed statistically significant correlation with the climatic parameter were used to build the subsequent models of inference (regressions) for that parameter.

Furthermore, another frequent problem arises when two or more of the independent variables are highly correlated to one another, which is called multicollinearity and causes inaccurate model parameterization, decreased statistical power, and exclusion of significant predictor variables during model development [98, 100, 101]. Therefore, when there are many possible explanatory variables, in order to obtain a parsimonious regression model it is necessary to reduce the number of variables by excluding redundant variables. To avoid problems with multicollinearity, several approaches have been suggested [100, 102]. On the one hand, the principal component analysis (PCA), including all the independent variables is habitually used because it removes all collinearity among the resulting components, which can then be used as independent variables in a multivariate regression [100]. Nevertheless, in many palaeoecological studies the most relevant components computed in the PCA are usually related to temporal or biogeographical factors, while those related to climatic variables are relegated to secondary positions with lower explanatory power of the community structure [24, 32, 35, 53, 79]. Therefore, we decided to follow an approach based on variable selection before computing a multiple regression for each climatic model [103, 104]. We computed correlations between all the body-size structure categories and, for each subsequent climate regression model, we removed one of the independent variables for each pair of body size categories that are significantly correlated (r > 0.5) between them. Finally, since multiple regression is not good at explaining the relationship of the independent variables to the dependent variables if those relationships are not linear [98], we also followed a bivariate approach in which we performed linear, quadratic and exponential regressions (following [32]) taking into account as independent variable only the body size categories that previously showed significant correlation with climatic variables.

Additionally, due to the presence of dissimilar body size community structures in faunas from different continents [53, 105], we set different models taking into account not only all the Old World localities together, but also taking separately localities from Asia or Africa. Therefore, all the procedures described above were repeated for each of these three data subsets.

**Fig 7:**
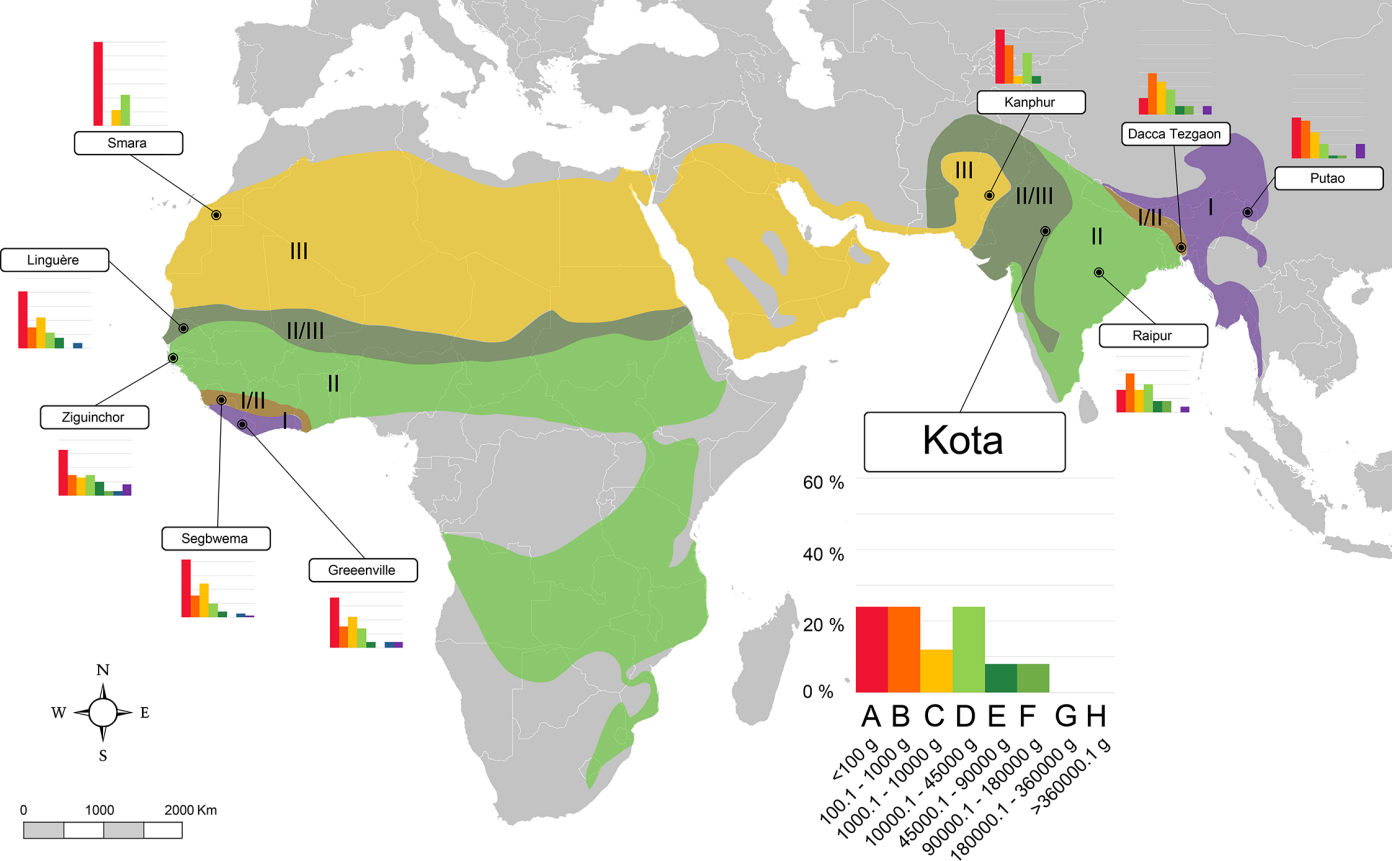
Map of the Palaeotropics showing the localities used in this work for validation of the palaeoclimatic quantitative models and the body-size structure of each one. Biomes as in Fig 2. A–H, body size categories as in Table 1. Data for each locality are shown in S4 Table.

Of all the regression models generated, only those with a determination coefficient (r^2^) higher than 0.5 were considered for validation. In order to validate these regression models, we applied them to ten new modern faunas (Fig 7), which differed from the previously used to calculate the regressions. We emphasized the inclusion of mammal assemblages from areas other than the previously sampled and from climatic conditions ranging from desert (III) to equatorial evergreen forest (I), both in Sub-Saharan Africa and South Asia. Faunal and climatic data on these localities are shown in S4 Table.

To test the accuracy of the regression models we followed the methodology of Hernández Fernández & Peláez-Campomanes [29]. In each case a coefficient of determination between the observed and predicted values for the climatic values in the new localities was calculated (r^2^_*p*_). These r^2^_*p*_ were compared to the coefficient of determination of the corresponding calculated regression (r^2^_*r*_) in order to determine the degree of decrease in goodness of fit. Due to sample chance, small decreases are expected even within accurate models. Hernández Fernández & Peláez-Campomanes [29] and Hernández Fernández & Vrba [32] considered a decrease in goodness of fit of 10% as the threshold for accepting a model as inaccurate. Thus, if r^2^_*r*_ − r^2^_*p*_ was larger than r^2^_*r*_/10 the model was considered inaccurate. Finally, if r^2^_*r*_ − r^2^_*p*_ was between r^2^_*r*_/10 and r^2^_*r*_/20 the model was considered to be accurate and, if it was lower than r^2^_*r*_/20, very accurate [29].

Finally, all the models that we considered accurate were applied to data from the Somosaguas fossil fauna in order to obtain climatic values for this Miocene fossil site. When more than one data subset gave accurate models for one climatic factor, we calculated the average of their climatic inferences.

All the statistical analyses were performed with SPSS v. 22.0. [106].

## Results

### Correlation of body size categories with climate factors

In the analysis including all the localities, 26 statistically significant correlations were found between climatic and body size variables (Table 3), and most of the body size categories showed significant correlations with several climatic variables. Only the largest body size category (H), which is constituted by megaherbivores (≥ 360 Kg), did not correlate with any of the climatic variables. There are very few species included in this category (mainly rhinoceroses, elephants and some ruminants), and the variability of their numerical relevance within communities does not appear to be related to climatic variables.

**Table 3:** Correlations between species percentage in each body size category and the values of climatic variables for the three data sets.

	Palaeotropics											
^a^\^b^	T (°C)		Tmin (°C)		Tmax (°C)		Mta (°C)		P (mm)		D (months)	
	r	p	r	p	R	p	r	p	r	p	r	p
A	**−0.589** ^c^	**<0.001**	**−0.270**	**0.032**	**−0.610**	**<0.001**	−0.184	0.150	**−0.265**	**0.036**	−0.084	0.512
B	**0.512**	**<0.001**	0.122	0.340	**0.653**	**<0.001**	**0.337**	**0.007**	0.240	0.058	0.120	0.348
C	0.124	0.332	**0.534**	**<0.001**	**−0.325**	**0.009**	**−0.666**	**<0.001**	**0.421**	**0.001**	**−0.384**	**0.002**
D	0.172	0.178	**−0.304**	**0.015**	**0.572**	**<0.001**	**0.640**	**<0.001**	−0.203	0.110	**0.392**	**0.001**
E	**0.323**	**0.010**	**0.439**	**<0.001**	0.015	0.907	**−0.358**	**0.004**	−0.054	0.676	−0.081	0.529
F	**0.354**	**0.004**	0.081	0.529	**0.480**	**<0.001**	**0.255**	**0.044**	0.141	0.270	−0.006	0.963
G	**−0.585**	**<0.001**	**−0.384**	**0.002**	**−0.546**	**<0.001**	−0.045	0.725	**−0.303**	**0.016**	0.081	0.527
H	−0.057	0.658	−0.071	0.583	−0.055	0.668	0.022	0.864	−0.037	0.776	0.010	0.939
	Africa											
	T (°C)		Tmin (°C)		Tmax (°C)		Mta (°C)		P (mm)		D (months)	
	r	p	r	p	R	p	r	p	r	p	r	p
A	**−0.531**	**0.001**	**−0.574**	**<0.001**	−0.301	0.075	**0.481**	**0.003**	0.028	0.872	−0.156	0.365
B	**0.394**	**0.017**	**0.488**	**0.003**	0.185	0.279	**−0.455**	**0.005**	0.329	0.050	−0.184	0.282
C	**0.376**	**0.024**	**0.507**	**0.002**	0.057	0.741	**−0.560**	**<0.001**	**0.344**	**0.040**	−0.258	0.129
D	−0.260	0.125	**0.367**	**0.028**	0.021	0.902	**0.447**	**0.006**	**−0.431**	**0.009**	**0.443**	**0.007**
E	**0.521**	**0.001**	**0.567**	**<0.001**	0.261	0.124	**−0.499**	**0.002**	0.059	0.734	−0.027	0.878
F	0.048	0.779	0.175	0.307	−0.063	0.716	−0.247	0.146	0.006	0.974	0.000	1,000
G	**−0.509**	**0.002**	**−0.746**	**<0.001**	−0.100	0.560	**0.814**	**<0.001**	**−0.394**	**0.017**	0.304	0.072
H	0.186	0.276	0.192	0.262	0.114	0.508	−0.152	0.375	−0.170	0.322	0.139	0.419
	Asia											
	T (°C)		Tmin (°C)		Tmax (°C)		Mta (°C)		P (mm)		D (months)	
	r	p	r	p	R	p	r	p	r	p	r	p
A	−0.309	0.116	−0.211	0.291	−0.091	0.653	0.125	0.536	−0.321	0.102	0.358	0.067
B	0.228	0.252	0.087	0.666	0.120	0.552	−0.013	0.948	0.029	0.885	0.230	0.249
C	0.083	0.679	**0.622**	**0.001**	**−0.584**	**0.001**	**−0.764**	**<0.001**	**0.714**	**<0.001**	**−0.628**	**<0.001**
D	0.117	0.560	−0.358	0.067	**0.587**	**0.001**	**0.557**	**0.003**	**−0.403**	**0.037**	0.307	0.119
E	0.343	0.080	0.203	0.309	0.211	0.290	−0.062	0.757	−0.109	0.588	−0.144	0.474
F	0.161	0.422	0.116	0.565	0.080	0.691	−0.054	0.788	0.010	0.960	−0.292	0.140
G	n.a. ^d^	n.a.	n.a.	n.a.	n.a.	n.a.	n.a.	n.a.	n.a.	n.a.	n.a.	n.a.
H	**−0.392**	**0.043**	**−0.464**	**0.015**	−0.084	0.679	0.328	0.094	0.122	0.545	−0.227	0.254

^a^ body size categories A–H as in Table 1

^b^ climatic variables; T, mean annual temperature; Tmin, mean temperature of the coldest month; Tmax, mean temperature of the warmest month; Mta, mean annual thermal amplitude; P, annual total precipitation; D, drought length

^c^ significant correlations are in bold

^d^ n.a., not available

In African communities, 21 correlations between body size categories and climate factors were significant. Temperature and thermal seasonality seemed to be the most relevant factors for the body-size structure of the African mammal communities (Table 3).

Among Asian modern faunas, only 10 significant correlations were found (Table 3). The body size category including species of 1–10 Kg (C) is highly correlated with most of the climatic variables. While the body size category D (10–45 Kg) was significantly correlated to temperature and precipitation, the body size category H (≥ 360 Kg) seemed to be related in some way to temperature, but with a low level of significance.

### Development of quantitative inference models

As expected, there were moderate to high levels of multicollinearity among body size variables in all the three data subsets (Table 4), which suggested the need to reduce the number of variables implied in each multiple regression model. Eventually, all the possible OLS multiple linear regression models (18), including only body size categories significantly correlated with the modelled climatic factor that were not correlated among them, were statistically significant (S5 Table). Nevertheless, these analyses showed only 6 multiple regression models that explained more than 50% of the variability in the original data.

**Table 4:** Correlations between body size categories for the three data sets.

	Palaeotropics															
Body size categories ^a^	A		B		C		D		E		F		G		H	
	r	p	r	p	r	p	r	p	r	p	r	p	r	p	r	p
A	1		**−0.648**^**b**^	**<0.001**	−0.173	0.168	−0.457	<0.001	−0.203	0.105	**−0.638**	**<0.001**	**0.634**	**<0.001**	-0.121	0.338
B	**−0.648**	**<0.001**	1		−0.195	0.120	0.307	0.013	−0.108	0.392	0.354	0.004	**−0.738**	**<0.001**	-0.175	0.162
C	−0.173	0.168	−0.195	0.120	1		−0.319	0.010	0.129	0.307	−0.253	0.042	−0.098	0.438	-0.153	0.223
D	−0.457	<0.001	0.307	0.013	−0.319	0.010	1		−0.443	<0.001	0.450	<0.001	−0.247	0.047	-0.323	0.009
E	−0.203	0.105	−0.108	0.392	0.129	0.307	−0.443	<0.001	1		0.144	0.253	−0.095	0.449	0.479	<0.001
F	**−0.638**	**<0.001**	0.354	0.004	−0.253	0.042	0.450	<0.001	0.144	0.253	1		−0.479	<0.001	0.167	0.183
G	**0.634**	**<0.001**	**−0.738**	**<0.001**	−0.098	0.438	−0.247	0.047	−0.095	0.449	−0.479	<0.001	1		0.069	0.582
H	−0.121	0.338	−0.175	0.162	−0.153	0.223	−0.323	0.009	0.479	<0.001	0.167	0.183	0.069	0.582	1	
	A	B	C	D	E	F	G	H
	r	p	r	p	r	p	r	P	r	p	r	p	r	p	r	p
A	1		−0.180	0.36	−0.298	0.123	**−0.505**	**0.006**	−0.316	0.101	−0.458	0.014	n.a.	n.a.	-0.096	0.625
B	−0.180	0.360	1		0.006	0.974	−0.214	0.275	−0.229	0.241	**−0.581**	**0.001**	n.a.	n.a.	-0.241	0.216
C	−0.298	0.123	0.006	0.974	1		−0.302	0.119	−0.172	0.381	−0.048	0.809	n.a.	n.a.	-0.321	0.096
D	**−0.505**	**0.006**	−0.214	0.275	−0.302	0.119	1		0.059	0.764	0.462	0.013	n.a.	n.a.	-0.063	0.752
E	−0.316	0.101	−0.229	0.241	−0.172	0.381	0.059	0.764	1		0.472	0.011	n.a.	n.a.	0.235	0.228
F	−0.458	0.014	**−0.581**	**0.001**	−0.048	0.809	0.462	0.013	0.472	0.011	1		n.a.	n.a.	0.147	0.455
G	n.a.^c^	n.a.	n.a.	n.a.	n.a.	n.a.	n.a.	n.a.	n.a.	n.a.	n.a.	n.a.	n.a.	n.a.	n.a.	n.a.
H	−0.096	0.625	−0.241	0.216	−0.321	0.096	−0.063	0.752	0.235	0.228	0.147	0.455	n.a.	n.a.	1	
	A	B	C	D	E	F	G	H
	r	p	r	p	r	p	r	P	r	p	r	p	r	p	r	p
A	1		−0.143	0.398	**−0.711**	**<0.001**	0.267	0.111	**−0.624**	**<0.001**	−0.155	0.361	0.406	0.013	-0.412	0.011
B	−0.143	0.398	1		0.101	0.551	−0.425	0.009	0.393	0.016	0.036	0.831	**−0.682**	**<0.001**	-0.116	0.493
C	**−0.711**	**0**	0.101	0.551	1		−0.161	0.343	0.187	0.268	−0.241	0.150	**−0.516**	**0.001**	-0.103	0.544
D	0.267	0.111	−0.425	0.009	−0.161	0.343	1		**−0.707**	**<0.001**	−0.411	0.012	0.308	0.064	**-0.552**	**<0.001**
E	**−0.624**	**<0.001**	0.393	0.016	0.187	0.268	**−0.707**	**<0.001**	1		0.380	0.020	−0.384	0.019	**0.595**	**<0.001**
F	−0.155	0.361	0.036	0.831	−0.241	0.150	−0.411	0.012	0.380	0.020	1		−0.118	0.488	**0.614**	**<0.001**
G	0.406	0.013	**−0.682**	**<0.001**	**−0.516**	**0.001**	0.308	0.064	−0.384	0.019	−0.118	0.488	1		0.007	0.965
H	−0.412	0.011	−0.116	0.493	−0.103	0.544	**−0.552**	**<0.001**	**0.595**	**<0.001**	**0.614**	**<0.001**	0.007	0.965	1	

^a^ categories A–H as in Table 1

^b^ significant correlations with r > 0.5 are in bold

^c^ n.a., not available

None of the multiple regression models was adequate for the inference of mean annual temperature or drought period (S5 Table). Nevertheless, appropriate models for the inference of mean temperature of the coldest and warmest month were derived from the dataset including all the localities (S5 Table). Additionally, mean annual thermal amplitude was properly modelled from data in all the three geographical sets (Palaeotropics, Africa and Asia) (S5 Table). Finally, the only significant inference model for total annual precipitation was derived from Asian faunas (S5 Table).

Not all the body size categories were similarly useful for climatic inference in each model. While in Africa the more predictive variables were A, D and G, in Asia they were C and D. And finally, for the whole dataset only the categories A and H were not useful for all the acceptable inference models (S5 Table).

For all the bivariate models with r^2^ > 0.5, the quadratic regression usually obtained the best adjustment, except for the regression of thermal amplitude in Asia, in which the best model derived from an exponential regression (S5 Table). These bivariate models showed better fit (higher values of r^2^) to the data than the multiple regression models in the case of the Asian communities but not for the African or the Palaeotropical datasets (S5 Table).

Most of the 21 models that reached the threshold of r^2^ > 0.5 were considered very accurate (r^2^_*r*_ − r^2^_*p*_ < r^2^_*r*_/20), but four of them were considered inaccurate (r^2^_*r*_ − r^2^_*p*_ > r^2^_*r*_/10) and, therefore, rejected (Table 5). Finally, we were able to find accurate bivariate models for the inference of mean temperature of the coldest month (Tmin, based on African faunas), mean annual thermal amplitude (Mta, based on African and Asian faunal subsets), as well as total annual precipitation and drought length (P and D, based on Asian faunas), from which climate inferences for the Somosaguas assemblage could be drawn. Only one multivariate model was accurate for the inference of a climatic variable (Mta, Palaeotropical subset).

**Table 5:** Inferences for climatic variables for the middle Miocene mammalian assemblage from Somosaguas.

Quantitative models for climatic inference ^a^								Climatic inference ^g^ for Somosaguas		
Number	Climatic factor ^b^	Continent	Body-size categories ^c^	model ^d^	r^2^_r_ ^e^	r^2^_p_ ^f^	r^2^_r_ − r^2^_p_	Inferences	Confidence Interval	Mean inference ^h^
1	Tmin (°C)	Palaeotropics	C,D,E,G	L	0.509	0.247	0.262	−		26.8
2		Africa	G	L	0.556	0.922	−0.366	25.1	17.5 − 32.6	
3			G	Q	0.579	0.942	−0.363	**26.8**	**18.9 − 34.8**	
4			G	E	0.567	0.938	−0.371	25.7	17.0 − 38.8	
5	Tmax (°C)	Palaeotropics	B,C,D,F	L	0.583	0.449	0.134	−	
6		Asia	C	Q	0.528	0.337	0.191	−	
7	Mta (°C)	Palaeotropics	B,C,D,E,F	L	0.633	0.681	−0.048	**8.4**	**5.4 − 11.4**	9.2
8			C	Q	0.522	0.534	−0.012	16.7	7.8 − 25.6	
9			C	E	0.505	0.533	−0.028	14.2	4.6 − 43.9	
10		Asia	C,D	L	0.632	0.929	−0.297	13.7	8.2 − 19.2	
11			C	L	0.584	0.990	−0.406	18	8.9 − 27.1	
12			C	Q	0.584	0.984	−0.4	18.1	8.6 − 27.7	
13			C	E	0.698	0.882	−0.184	**19.0**	**8.9 − 40.7**	
14		Africa	A,D,G	L	0.733	0.132	0.601	−		
15			G	L	0.663	0.713	−0.05	0.9	−4.7 − 6.5	
16			G	Q	0.666	0.721	−0.055	**0.3**	**− 5.7 − 6.3**	
17			G	E	0.644	0.665	−0.021	2.0	0.8 − 5.3	
18	P (mm)	Asia	C,D	L	0.516	0.613	−0.097	610.8	− 378.4 − 1600.0	219.3
19			C	L	0.510	0.537	−0.027	389.6	− 1149.5 − 1928.6	
20			C	Q	0.529	0.585	−0.056	**219.3**	**− 1365.1 − 1803.7**	
21	D (months)	Asia	C	Q	0.508	0.850	−0.342	**10.3**	**6.2 − 14.3**	10.3

^a^ according to the different regression models presented in S5 Table

^b^ as in Table 3

^c^ categories A–H as in Table 1

^d^ L, linear regression models; Q, quadratic regression models; E, exponential regression models

^e^ coefficient of determination of the quantitative bioclimatic models

^f^ coefficient of determination of the regression between the observed and predicted values

^g^ only for accurate models (mean ± 95% confidence interval); inference of the best fitting model for each climate parameter in each data subset is shown in bold

^h^ mean of the best fitting models for each climate parameter in each data subset

### Climatic inference for the Somosaguas assemblage

**Fig 8:**
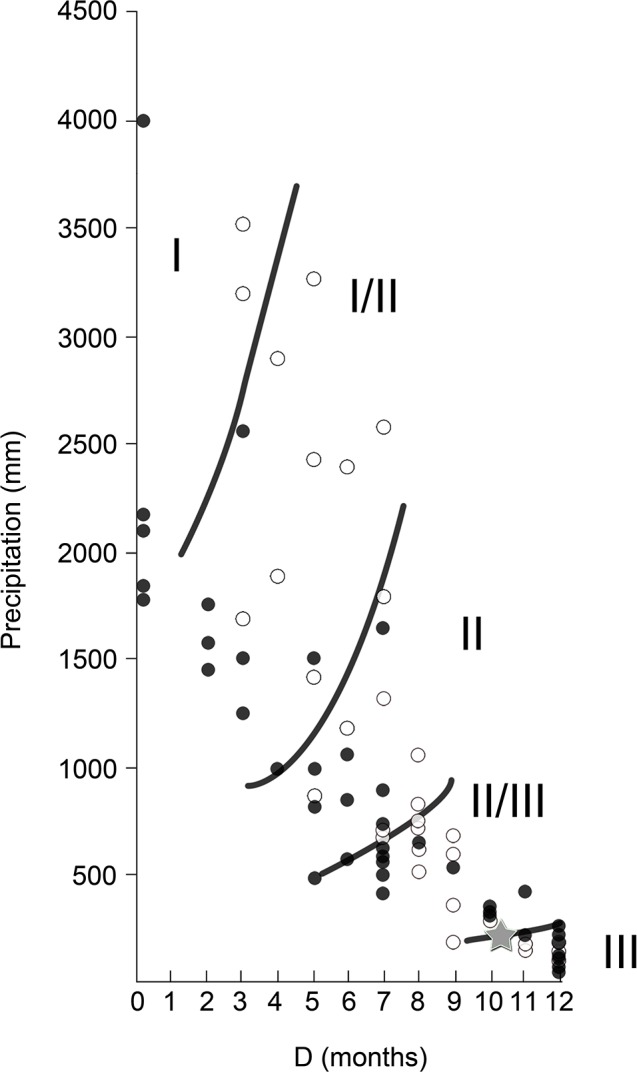
Mammalian body-size structure of the Somosaguas fossil site. Categories as in Table 1.

Table 5 shows the inferences for the Somosaguas assemblage, according to its body-size structure (Fig 8), obtained for each climatic parameter in all the accurate regression models. In each data subset (Palaeotropic, Africa and Asia) we selected the best fitting model for each climatic factor. Then, when accurate models for each climatic factor were derived from different datasets, we computed the average of their climatic inferences (Table 5).

**Fig 9:**
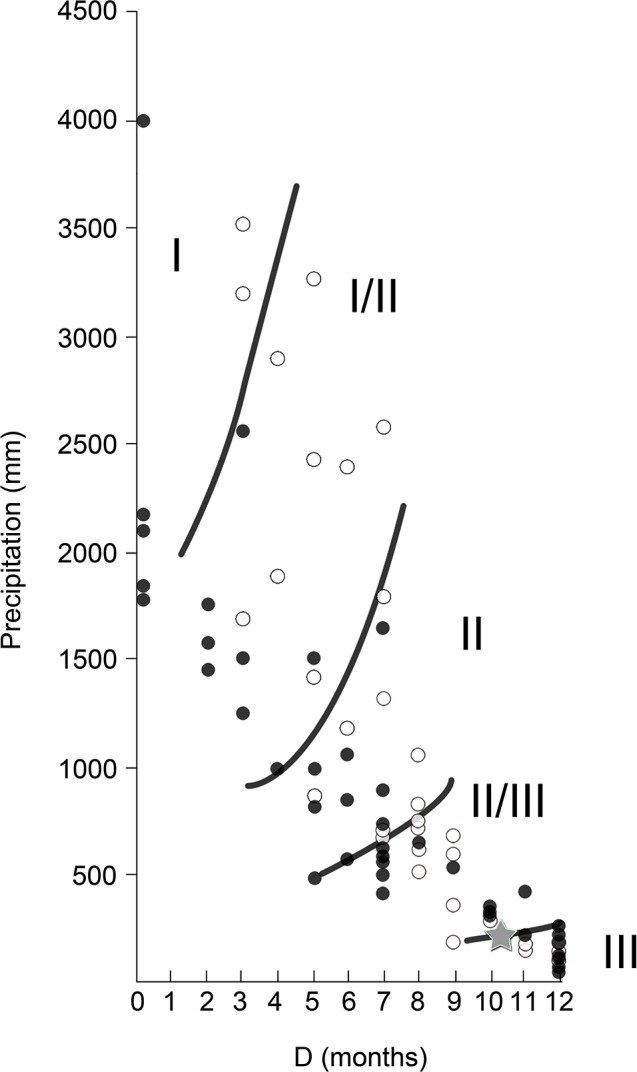
Relationship between total annual precipitation (P) and drought length (D) for data from all the localities from Asia (white) and Africa (black) studied in this work. Values for the Somosaguas fossil site are shown with a star. Limits between biomes (I, evergreen tropical rain forest; I/II, semi-evergreen tropical forest; II, tropical deciduous woodland; II/III, savanna; III, subtropical desert) are from Walter [36].

Finally, our results showed climate conditions that are congruent with environments in the ecotonal zone between the savanna (II/III) and desert (III) biomes, with around 10 months of drought length and an annual total precipitation higher than 200 mm per year (Fig 9), although confidence intervals for the climatic predictions are relatively wide (Table 5).

## Discussion

### Climatic inference models

Methods of quantitative palaeoclimatic inference are very scarce due to the difficulties associated with the calculation of adequate numerical models from modern faunal data, since ecological structure of mammalian faunas is not only dependent on environmental factors, but also on other aspects such as biogeography and history [84, 107–110]. Our method takes separate approaches including different faunal data sets and, therefore, it reduces the variability due to historical and biogeographic factors, which in other analyses limits the power of palaeoclimatic inferences. Furthermore, our taxon-free approach also avoids the error of assuming the ecological preferences of an extinct animal in comparison with the ecological preferences of the more closely related living species. Particularly, due to the loss of ecological diversity, inference of the habitats of extinct species of diversified groups in the past that are today represented only by relics might be not reliable, as pointed out by Van Couvering [111]. This could be the case for some of the Somosaguas mammals that have been usually interpreted as forest dwellers. For example, *Dorcatherium crassum* was a very large tragulid (around 25–30 Kg according to Alba et al. [86]), which makes it a substantially different species from an ecological point of view when compared to its modern relatives, whose body mass is around 10 Kg. Large species usually have larger geographical distributions and are able to inhabit several different biomes [109]. Additionally, the remains of this species in Somosaguas are very scarce, which might suggest it was rare in such an arid area, and was probably associated with the most humid environments and even then only present concomitant with especially humid years. Another taxon that is usually assumed to be a forest dweller is *Micromeryx*, but other authors have pointed out that their diversification in the Iberian Peninsula gave rise to different lineages, sometimes with wide ecological preferences [88].

The proportion of species in the body size category G (mammals from 180 to 360 Kg) is indicative of the mean temperature of the coldest month and thermal seasonality in African faunas. These climatic variables are related to the degree of vegetation coverage in the Paleotropics, following a gradient from cooler and more seasonal open environments to warmer and less seasonal tropical forests; usually the former have higher number of large species [19]. Emmons et al. [112] also related large (<100 Kg) African mammals with dry lands. This appears to be related to the extraordinary diversification undergone by African mammals from open environments (especially ruminants, many of them included in the G category) associated with the global cooling related to the Northern Hemisphere glaciations [107, 113].

The proportion of species in the body size category C (involving mammals from 1 to 10 Kg) was found to be the best proxy for the aridity-humidity gradient when using southeastern Asian mammal faunas as ecological analogues. This body size category in Asia is clearly dominated by species associated with humid and arboreal habitats, such as primates, pangolins, and some rodents and artiodactyls. Therefore, the arid localities have lower species in the C category and, conversely, the humid localities have numerous species belonging to the C body-size category.

### Potential flaws of the models

The determination coefficients (r^2^) calculated for most of the models presented here were lower and the confidence intervals wider than in other similar quantitative palaeoclimatic studies of Plio-Pleistocene faunas [29, 32]. Nevertheless, such models are based on other kinds of community structure data (e.g. bioclimatic or taxonomical structures), in which the relationships between climate variables and modern faunas are difficult to extrapolate to the middle Miocene faunas. Miocene mammalian communities show many historical, biogeographic and evolutionary particularities when compared to the modern reference faunas used as ecological analogues for the computation of statistical models. Additionally, modern faunas from different continents are also the product of different contingent histories. All this may preclude the use of models that strongly depend on the maintenance of the variables used for the establishment of community structure between certain values of similarity with modern faunas (e.g. taxonomic structure). Our taxon-free approach may have partially resolved this problem.

Although there are methods for the description of community structures that are robust against sampling or taphonomical biases [114, 115], our quantitative method appears to be sensitive to relatively small changes in the community structure. Therefore, this method should only be applied to well sampled fossil sites in which taphonomical biases have not erased the ecological signal. In this sense, the taphonomic study of this fossil site has not detected a significant bias in any part of the body-size structure, as it includes species across all the body size spectra [63, 116, 117]. Additionally, Somosaguas is a rich, diverse and intensively excavated site with an exceptional number of fossil remains [46, 117], which guarantees a high level of confidence about the identification of species recorded in it. In fact, a great proportion of the total fauna present in the middle Miocene of the Madrid Basin has been already described in Somosaguas [118]. Finally, since we only used presence of species, our approach is not influenced by potential biases that may affect the relative abundance of their remains [63].

Another potential limitation of our approach is the possible existence in the past of climatic combinations unknown today. Nevertheless, global Neogene climate dynamic seems to have been substantially similar to the one observed today [119–121].

### Middle Miocene climate of Somosaguas

During the middle Miocene, between 17 and 15 Ma, the temperatures increased [2] in what is known as the Miocene Climatic Optimum (MCO), when the climate was warm and humid and the tropical belt had an expanded latitudinal extent [122]. Around 14.8 Ma a cooling process started, associated with the increase of the Antarctic ice sheet, and both thermal and hydric seasonality increased. Although, this trend continued slowly until the end of the Miocene in a process known as Middle Miocene Global Cooling Event, the major part of the cooling occurred in less than a million years, resulting in a rapid shift from relative high-latitude warmth to high latitude refrigeration [123]. As a consequence of this change, the latitudinal temperature gradient increased, which fortified climatic frontiers and increased aridity in middle latitudes [124]. The Alpine orogeny and the formation of the distant Tibetan Plateau also provoked a decrease of mean annual temperature and precipitation in Europe [124–127]. All these changes were also reflected in the Iberian Peninsula [128–131]. Aridity peaks have been described over the middle Aragonian of Spain based on the mammalian record [21, 35] and particularly in the local biozone E [13], which includes the Somosaguas fossil site. Furthermore, previous works based on isotopic data of herbivore dental enamel [12, 13] correlated the aridity increase detected in this biozone with the Middle Miocene Global Cooling event in Somosaguas [3]. Other studies based on different characteristics of the mammalian community of Somosaguas such as Hernández Fernández et al. [24] and Perales et al. [47] identified the mammalian association from the middle Miocene of Somosaguas as a savanna community, and García Yelo et al. [39] classified it as an arid to semiarid environment. Our results, which indicate a transitional ecotone environment between savanna and desert are, therefore, congruent with the cited previous studies.

Although the confidence intervals of our estimations were wide and we have to be cautious with the results, we compared our inferences with previous works. We were not able to compute a valid model for the inference of mean annual temperature in order to compare it with previous results provided by Domingo et al. [12] for the variation in mean annual temperature (T) based on isotopic data (from a maximum value of 26.6°C in level T1 of Somosaguas to 11.6°C in T3). Nevertheless, our inference for mean temperature of the coldest month (Tmin, 26.8°C ± 8) indicated higher values than expected, considering that Tmin should be lower than T.

The regression models computed for the inference of mean thermal amplitude gave very different results depending on the continent from which the reference faunas were taken (Table 5). This could be partially related to the different evolutionary history of mammals from different continents, with an important radiation of Plio-Pleistocene large ruminants (G category) in the African continent associated with the global increase of seasonality, while Asian faunas show a higher relationship of this climatic parameter with smaller mammal species (C category). Also, variations in seasonality associated with the Middle Miocene Cooling Event [3] could be hampering correct inferences of thermal amplitude during the middle Miocene of the Iberian Peninsula.

Our results mostly support previous estimates of seasonality of moisture in Somosaguas based on clay minerals [48]. Although our analyses yield an estimate of 10.3 months of drought and 219 mm of total annual precipitation, Carrasco et al. [48] reported values of 8–9 months and 100–500 mm respectively based on data from clay minerals. Additionally, isotopic analyses by Domingo et al. [13] calculated mean annual precipitation values around 400 mm for the local biozone E in the Madrid Basin. Although this value is higher than our inference, it still would place Somosaguas among the most arid savannas, very close to the ecotone with tropical deserts. Similar palaeoprecipitations were obtained by Böhme et al. [132] in the early middle Miocene of the Calatayud-Daroca Basin of northern Spain (130–321 mm). Our results were also congruent with the arid climate inferred in south-central Spain during the middle Miocene [26].

Additional information can be derived from the recent work on the taphonomy of the Somosaguas fossil site by Domingo et al. [117]. These authors found weathering traces in the fossils, which indicate that bones were exposed for varied time, ranging from one year until a minimum of four years before a debris-flow accumulated them. This indicates high levels of interannual variability in the precipitation regime, which is in agreement with the highly erratic nature of precipitations in arid tropical areas today [133, 134, 135]. Long drought periods associated with such scarce and erratic rainfalls have been held responsible for the high mortality levels of young individuals recorded in the Somosaguas fossil site for different species [45, 117, 136–139].

### Vegetation and landscape structure

Our results suggest that the Miocene fauna from Somosaguas inhabited an ecotonal zone between savanna (II/III) and the tropical desert biome (III). Under these climatic conditions modern day vegetation is dominated by semidesert formations, which are characterized by adaptations to arid climates [36]. This is congruent with the communities of leguminous plants that dominated the landscape of many Iberian regions from the Oligocene to the middle Miocene, including species of the genera *Acacia*, *Albizia*, *Caesalpinia*, *Cassia*, *Hylodesmum*, *Mimosa* and *Gleditsia* [140]. Unfortunately, there is a general lack of plant or pollen fossil localities in the interior of the Iberian Peninsula for the middle Miocene [141]. Only two sites in the Madrid Basin are of roughly similar age to the Somosaguas fossil site and only one additional site in the Iberian Peninsula coincides in age.

The palaeobotanical assemblages, including both pollen and macrofossils, around the Culebro stream (Parla and Pinto, southern Madrid) described by Fernández Marrón et al. [142], Fernández Marrón et al. [143], Fernández Marrón et al. [144] are slightly younger than Somosaguas (MN 6). These fossil assemblages indicate a scanty, savanna-like vegetation dominated by xerophytic (adapted to arid environments), small-leaved shrubs and trees (eg. *Ephedra*, *Tetraclinis*, *Ziziphus*, *Pistacia*), including several with Palaeotropical ascendence within the families Celastraceae (*Celastrus*) and Fabaceae (*Robinia*, *Colutea*), while riparian and swamp environments were also present (e.g. *Taxodium*, *Populus*, *Alnus*, *Nyssa*, *Myrica*, *Sabal*, *Typha*, *Phragmites*).

Although climatic inferences suggest a colder and wetter climate, palynological data from the Aragonian of Puente de Toledo [140] indicate the development of open ecosystems dominated by herbaceous species in Poaceae and Asteraceae, and to a lesser degree in Amaranthaceae–Chenopodiaceae and Plumbaginaceae, with scarce representation of woody taxa such as *Quercus* or *Olea*. Riparian formations have been also recognized.

Finally, pollen-assemblage data from Gor (Granada, southeastern Spain) in samples dating 13.6–15.9 Ma indicate that herbs and shrubs dominated the middle-Miocene plant communities on the southern Iberian Peninsula, with grasses comprising 8–20% of terrestrial pollen abundance. Recent isotopic analyses of grass-pollen δ^13^C data indicated that the proportions of C_4_ species among grasses might range from 50 to 72% [145]. Nevertheless, although C_4_ grasses constituted substantial proportions of total grasses in Spain during the early-middle Miocene, they were not dominant in the ecosystems of Central Iberia, as revealed by isotopic analyses of tooth enamel in large herbivores [12, 13]. In any case, abundance of xerophytic subdesertic plants and C_4_ grasses indicates warm and dry conditions, and a seasonal climate where precipitation falls mostly during warm weather months [146], similar to the ones inferred for the Somosaguas fossil site.

**Fig 10:**
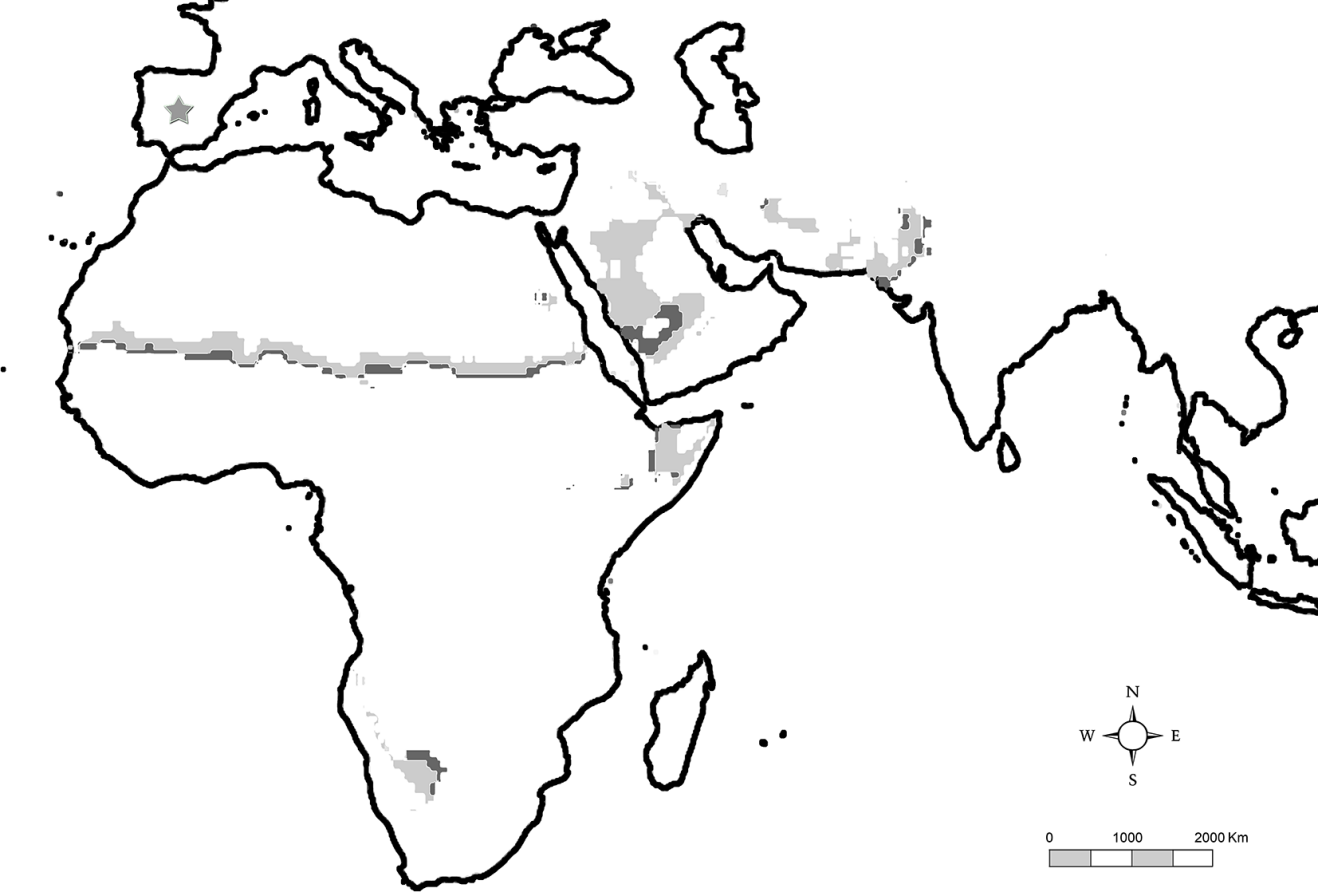
Map showing current regions in the Old World tropics and subtropics with similar values for both temperature and precipitation to those calculated for the Somosaguas fossil site (Table 5). The Somosaguas climatic values inferred for precipitation lay in areas placed at the edge limit between the light shaded region (125–224 mm precipitation) and the dark shaded region (225–274 mm). Current climatic distribution data were taken from Climate Research Group [160]. The geographic location of the Somosaguas fossil site is shown with a star.

Due to the paucity of palaeobotanical information on the landscapes of the Madrid Basin around 14 Ma, and following the concept of community convergence [71, 72] as well as the strong relationship between climate and vegetation physiognomy [36, 147], one reasonable source of information might be derived from the current vegetation in areas of the Old World with similar climatic characteristics to the ones inferred for the middle Miocene of Somosaguas, which can be found in the Sahel in North Africa, the Horn of Africa, the boundary zone between the Kalahari and the Namib in southern Africa, south-central Arabia, as well as in southern Pakistan and northeastern India (Fig 10). They can be defined as semi-desert areas, which are characterized by mean annual rainfall between 50–350 mm [37, 148, 149] and maintain a positive correlation between the total cover and the amount of annual precipitation, with around 50% cover when rainfall reaches 200 mm [149]. Nevertheless, although it does account for the vegetation performance of semideserts as a whole, annual precipitation is not the sole factor controlling the occurrence of different vegetation types in tropical and subtropical areas. There are varied and delicate functional interactions between several factors, which are of particular significance for the complex patch mosaic distribution of plant associations in semi-arid terrains [150–155]. Besides climatic factors, plant associations in semidesert areas depend on the interaction between soil physico-chemical composition, microtopography, geomorphology, capacity of water retention, and the effect of short-term events like fires or grazing [134, 135, 155–159].

Therefore, under the particular circumstances of climate in the arid regions depicted in Fig 10 the landscape consists of a patch mosaic of multiple plant associations, with a very marked zonation of vegetation [150]. Ecological succession and spatial transitions among plant communities depends upon biotic and abiotic disturbances as well as variations in local conditions matching geomorphological features of the landscape. These are usually associated with the soil water retention properties of the various substrates and micro-topographical variations in any particular area [149, 151, 161–164].

The Miocene sediments in Somosaguas indicate the presence of varied substrates in a landscape characterized by plains with gentle gradients, which were interrupted by depressions that received runoff water and waterborne fine materials [58, 59]. This topographic features and the tropical and arid climatic conditions inferred in this and previous works [12, 13, 24, 39, 47, 48, 117] could have supported a complex mosaic of vegetation dominated by open environments. This would combine zonal vegetation types (determined mostly by climate), such as spinous bushland and semidesert grassland, with azonal vegetation types (determined mostly by other parameters than climate) associated to drainage structures in the area, such as relatively luxuriant riverine communities in seasonal water flows or mudflats with xerophytic vegetation.

## Conclusions

We developed new models for quantitative palaeoclimatic inference based on regression analyses applied to the body-size structure of non-carnivore mammal associations. The independent use of faunal sets from tropical areas in Asia and Africa reduced high variability due to historical and biogeographical factors and facilitated the inference of several climatic variables. We present here a new analytical approach that could be applied to estimate palaeoclimate variables in other Miocene mammalian fossil sites from Southern Europe.

Although their confidence intervals were relatively wide, inference of temperature variables (mean temperature of the coldest month), precipitation and drought length for Somosaguas were congruent with previous results based on isotopic and mineralogical data. We also provided novel estimates for thermal seasonality (mean thermal amplitude). These were, however, very different depending on the modern dataset used to calculate them.

Palaeoclimatic quantitative inferences for the Somosaguas fossil site permitted a classification of the area during the Miocene as tropical arid, placing it in the dry savanna ecotone limiting with the tropical desert. These results are congruent with the increasing aridity inferred by previous studies for the middle Miocene of Southwestern Europe, related to the Middle Miocene Global Cooling Event. The environment of Somosaguas 14 millions of years ago was probably similar to the current environment of the Sahel region in North Africa, the Horn of Africa, the boundary area between the Kalahari and the Namib in Southern Africa, south-central Arabia, or eastern Pakistan and northwestern India. The vegetation of these areas shows a complex mosaic of plant communities, dominated by scattered xerophilous shrublands, semidesert grasslands and azonal vegetation linked to seasonal watercourses and ponds, which reflect a delicate adjustment to soil conditions related directly to geomorphology and to geology under the influence of unpredictable rainfall seasonality and climatic changes.

## Supporting information

S1 Table Modern African faunas used in this work, body size data and climatic variables for each locality. (XLSX)

S2 Table Modern Asian faunas used in this work, body size data and climatic variables for each locality. (XLSX)

S3 Table Climatic variables studied in this work. (XLSX)

S4 Table Modern Palaeotropical faunas used for models validation, body size data and climatic variables for each locality. (XLSX)

S5 Table Coefficients and their significance for the regression models for climatic inference based on the body-size structure of the three faunal datasets. (XLSX)
