# Correction: Body-size structure of Central Iberian mammal fauna reveals semidesertic conditions during the middle Miocene Global Cooling Event

**DOI:** 10.1371/journal.pone.0202612

**Published:** 2018-08-14

**Authors:** Iris Menéndez, Ana R. Gómez Cano, Blanca A. García Yelo, Laura Domingo, M. Soledad Domingo, Juan L. Cantalapiedra, Fernando Blanco, Manuel Hernández Fernández

Fig 8 is incorrectly duplicated from Fig 9. The authors have provided a corrected version of Fig 8 here.

**Fig 8:**
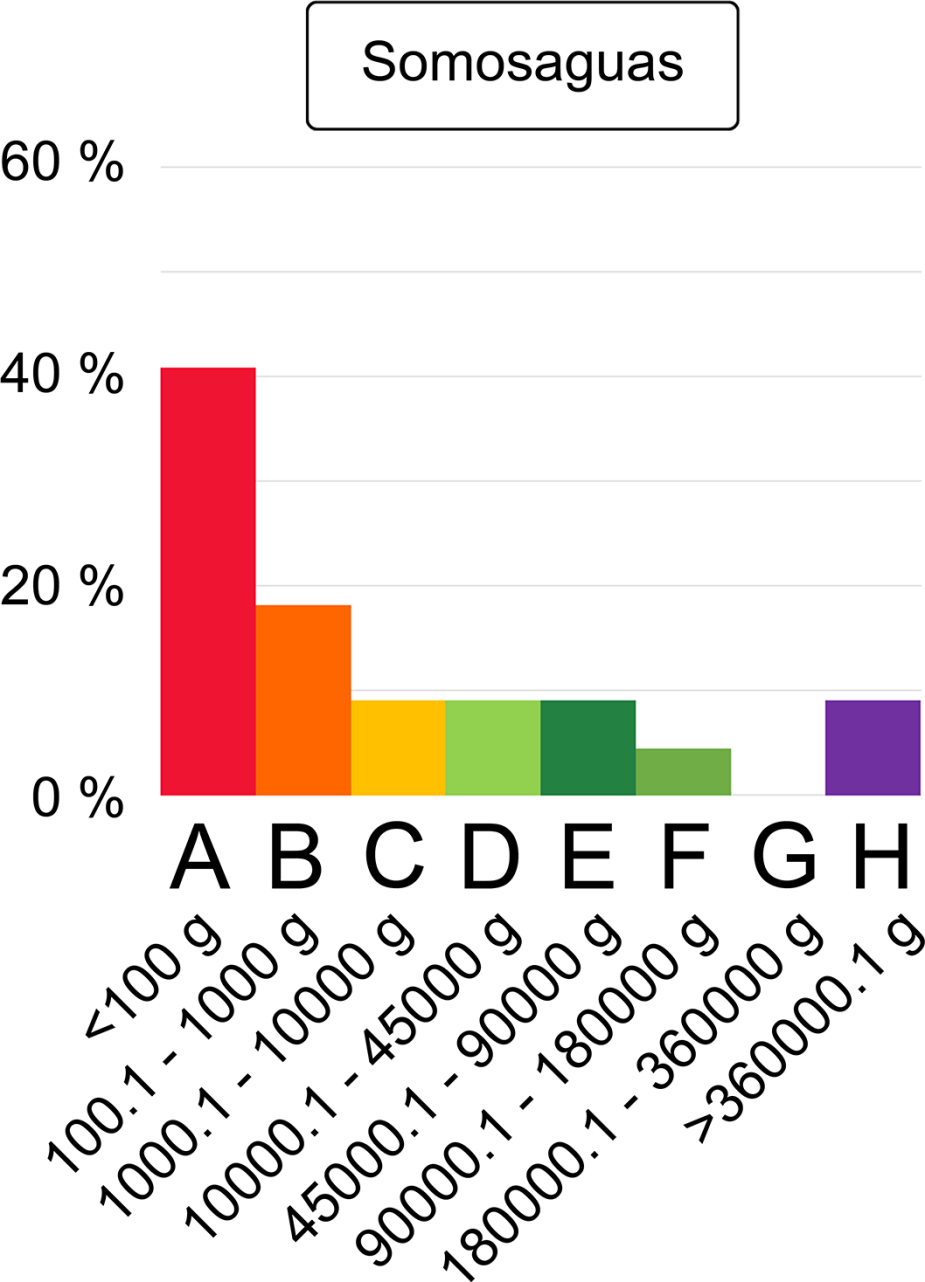
Mammalian body-size structure of the Somosaguas fossil site. Categories as in Table 1.
